# A Single Dose of Oral BCG Moreau Fails to Boost Systemic IFN-*γ* Responses to Tuberculin in Children in the Rural Tropics: Evidence for a Barrier to Mucosal Immunization

**DOI:** 10.1155/2012/132583

**Published:** 2012-01-11

**Authors:** Maritza Vaca, Ana-Lucia Moncayo, Catherine A. Cosgrove, Martha E. Chico, Luiz R. Castello-Branco, David J. Lewis, Philip J. Cooper

**Affiliations:** ^1^Centro de Investigaciónes FEPIS, Esmeraldas Quinindé, Ecuador; ^2^Vaccine Centre, St George's University of London, Cranmer Terrace, London SW17 ORE, UK; ^3^Laboratorio de Biologia Molecular, Hospital de Los Valles, Via Interoceanica Km 12.5, Cumbayá, Quito, Ecuador; ^4^Fundacao Ataulpho de Paiva, Avenida Pedro II, 226 Sao Cristovao, 20941-000 Rio de Janeiro, RJ, Brazil; ^5^Department of Immunology, Fundacao Instituto Oswaldo Cruz, 4365 Avenida Brasil, 21040-360 Rio de Janeiro, RJ, Brazil; ^6^Molecular and Biochemical Parasitology, Liverpool School of Tropical Medicine, Pembroke Place, Liverpool L3 5QA, UK; ^7^Colegio de Ciencias de la Salud, Universidad San Francisco de Quito, Via Interoceanica S/N y Avenida Pampite, Quito, Ecuador

## Abstract

Immune responses to oral vaccines are impaired in populations living in conditions of poverty in developing countries, and there is evidence that concurrent geohelminth infections may contribute to this effect. We vaccinated 48 children living in rural communities in Ecuador with a single oral dose of 100 mg of BCG Moreau RDJ and measured the frequencies of tuberculin-stimulated peripheral blood mononuclear cells expressing IFN-*γ* before and after vaccination. Vaccinated children had active ascariasis (*n* = 20) or had been infected but received short- (*n* = 13) or long-term (*n* = 15) repeated treatments with albendazole prior to vaccination to treat ascariasis. All children had a BCG scar from neonatal vaccination. There was no evidence of a boosting of postvaccination IFN-*γ* responses in any of the 3 study groups. Our data provide support for the presence of a barrier to oral vaccination among children from the rural tropics that appeared to be independent of concurrent ascariasis.

## 1. Introduction

The use of oral vaccines in children and adults from poor populations, particularly in the tropics, has been associated with reduced efficacy and impaired immune responses. Impaired immune responses have been reported for both live-attenuated and killed vaccines including Sabin oral polio vaccine [1], rotavirus [2, 3], cholera [4], *Shigella* [2, 5], and a killed *Vibrio cholerae* O1 plus B subunit vaccine [6]. Such effects are observed at doses that are highly immunogenic in subjects from North America or Europe and, in some circumstances, may be overcome by an increase in the size of vaccine dose or the number of doses administered [4, 7–9].

Barriers to effective vaccination with oral vaccines in poor populations living in the tropics include nutritional deficiencies (e.g., vitamin A and zinc), chronic diarrhoea, pre-existing mucosal immunity (e.g., intestinal secretory IgA), the presence of maternal antibodies in breast milk, environmental enteropathy [3, 4], and coinfections with enteric bacterial infections, and protozoal (e.g., *Giardia intestinalis*) and geohelminth parasites [10]. The geohelminth parasites, *Ascaris lumbricoides*, *Trichuris trichiura*, and hookworm have a worldwide distribution and are strongly associated with poverty. Geohelminths infect an estimated 2 billion humans [11] and prevalence, in the case of *A. lumbricoides*, is highest among preschool and school age children. Infections are chronic, tend to be acquired during the second year of life, and may persist into adulthood through residence in an environment that is heavily contaminated with human faeces. Geohelminth infections have also been associated with impairment of vaccine immune responses in experimental animal models [12] and in humans [13–15].

Oral BCG Moreau RDJ is a potent inducer of mucosal Th1-immune responses [16] and is registered for use in humans [17]. When given as a single dose to subjects with a BCG vaccination scar in the UK, oral BCG is able to boost IFN-*γ* responses to PPD [16]. Because ascariasis is associated with strong Th2 immune responses [18] systemically and at the site of infection in the intestinal mucosa [19], and Th1 and Th2 responses are considered to be reciprocally inhibitory [20]. We hypothesized that concurrent infections with ascariasis would strongly impair Th1 responses to tuberculin following vaccination with oral BCG, and that treatment with albendazole before vaccination would improve this response.

In the present study, we measured IFN-*γ* response to tuberculin *in vitro* before and after vaccination with a single dose of the live oral vaccine, BCG Moreau RDJ, in children who were either actively infected with *A. lumbricoides* or previously infected but had received either short- or long-term repeated treatments with the anthelmintic drug, albendazole, before vaccination.

## 2. Methods

### 2.1. Study Design and Subjects

**Figure 1:**
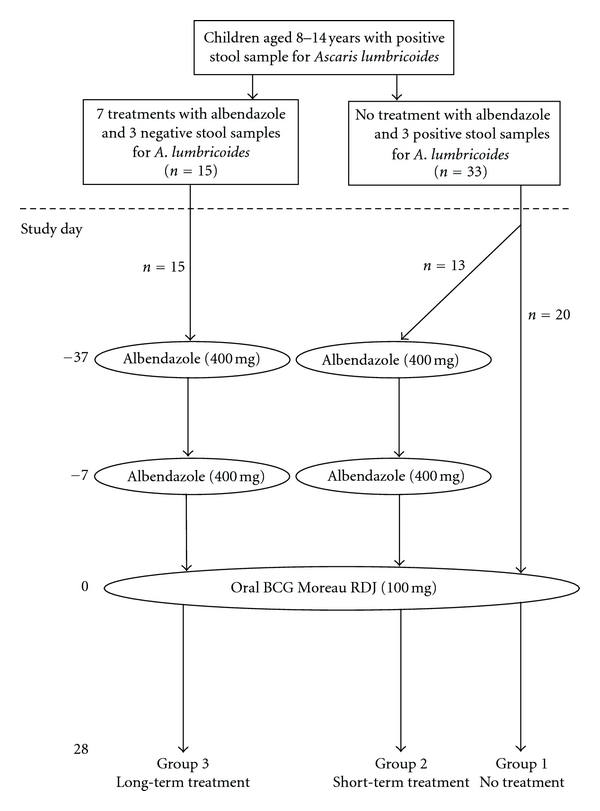
Study design. All eligible children had documented stool samples with *Ascaris lumbricoides* a year before the start of the study. Children were selected into study groups on the basis if they had or had not received 7 repeated doses of 400 mg of albendazole every 2 months over the previous 16 months. Children who had not received long-term albendazole and who continued to have ascariasis were allocated to 2 groups. Group 1: active *A. lumbricoides* infection; Group 2: short-term anthelmintic treatment. Children in Group 2 were given 2 single doses of 400 mg albendazole separated by 30 days. Children who had received long-term anthelmintic treatment were selected into Group 3. Children in Group 3 were given 2 single doses of 400 mg albendazole separated by 30 days. All children received a single dose of oral BCG Moreau RDJ. Blood was collected immediately before vaccination and at 28 days after vaccination to measure IFN-*γ* responses to PPD.

Children attending rural schools in the districts of Pedro Vicente Maldonado, Puerto Quito, and San Miguel de Los Bancos in a subtropical region of Pichincha Province were eligible. Subjects were selected from a database compiled by the study team for a cluster-randomized intervention study in which schools were randomized to receive either 400 mg of albendazole every 2 months for a year or no intervention [21]. All children in this intervention study received a single dose of 400 mg of albendazole at 12 months after the start. Eligible children were those aged 8–14 years, with a BCG scar on the upper right arm (parenteral BCG is given at birth in Ecuador), with no clinical evidence of immunodeficiency or current illness, and with results available from 3 previous stool examinations. Girls had a pregnancy test before inclusion. The study design is shown in Figure 1. Subjects were recruited into 3 groups. Group 1 (active infection with *A. lumbricoides*)—children with a current *A. lumbricoides* infection and 3 previous stool samples positive for *A. lumbricoides*. Group 2 (short-term anthelmintic treatment)—children with a current *A. lumbricoides* infection and 3 previous stool samples positive for *A. lumbricoides*. Group 3 (long-term anthelmintic treatment)—children who had received 7 previous doses of 400 mg of albendazole over the previous 16 months and had a positive stool sample for *A. lumbricoides* before the start of treatment but no positive stool samples for *A. lumbricoides* infections since the start of treatment. We compared short-versus long-term anthelmintic treatments because the immunological effects underlying putative vaccine hyporesponsiveness associated with ascariasis may require a significant time period of being parasite free to be reversed [10, 21, 22]. The study was conducted between January and May 2005. Informed written consent was obtained from a parent of each child. The study protocol was approved by the Ethics Committee of the Hospital Pedro Vicente Maldonado, Pedro Vicente Maldonado, Ecuador.

### 2.2. BCG Vaccination and Albendazole Treatments

Children in Groups 2 and 3 (short-term and long-term anthelmintic treatment, resp.) were given two directly observed doses of 400 mg of albendazole spaced 30 days apart with the last dose given 7 days before vaccination. Group 1 (active infection with *A. lumbricoides*) did not receive albendazole before vaccination. We used the oral BCG, still licensed for human use in Brazil, prepared from a World Health Organization, defined seed lot designated *Mycobacterium bovis* BCG substrain Moreau Rio de Janeiro by Fundacao Ataulpho de Paiva, Rio de Janeiro, Brazil. The BCG is cultured in a proprietary Sauton medium, suspended in 5 mL 1.5% sodium glutamate solution in a single 100-mg dose. The lot used in the present study was shown to contain 7.5–9.0 × 10^7^ cfu viable bacilli per 100 mg dose. All children were given a single oral dose of 100 mg of BCG Moreau RDJ re-suspended in 50 mL of 2% bicarbonate buffer as described previously [16]. A single dose of 400 mg of albendazole was given to all subjects at the end of the study.

### 2.3. Blood and Stool Samples

Blood samples (10 mL) were drawn into Vaccutainer tubes (Becton Dickinson) containing sodium heparin immediately before vaccination and 28 days after vaccination. Stool samples were collected from children in all study groups at the beginning of the study and (before albendazole treatment in Groups 2 and 3 and at the same time in Group 1) and at 28 days after vaccination. Stool samples were examined using the modified Kato-Katz (for quantification of egg counts) and formol-ether acetate concentration methods [23].

### 2.4. IFN-*γ* Enzyme-Linked Immunospot (ELISPOT) Assays

The frequencies of peripheral blood mononuclear cells (PBMCs) expressing IFN-*γ* were measured by ELISPOT as described previously [16]. Briefly, unfractionated PBMCs were separated from whole blood and were plated onto microtiter plates with nitrocellulose membranes (Millipore, Watford, UK). PBMCs were cultured for 18 hours in the presence of medium only, tuberculin (PPD; Statens Serum Institute, Copenhagen, Denmark) at 5 *μ*g/mL and phytohaemagglutinin (PHA; Sigma-Aldrich, Poole, UK) at 5 *μ*g/mL in RPMI 1640 medium supplemented with 10% fetal bovine serum, L-glutamine, gentamicin, and 1% HEPES in a humidified environment with 5% CO_2_ at 37°C. Anti-human IFN-*γ* antibody coating and detection antibody pairs and detection reagents for ELISPOT were used according the manufacturers recommendations (BD Biosciences, Oxford, UK). Spots were counted using an automated ELISPOT reader (AID Elispot Reader Systems, Strassberg, Germany). The background activity of IFN-*γ*-secreting cells in control wells was constant (data not shown).

### 2.5. Statistical Analysis

Results were expressed as frequencies of IFN-*γ*-expressing cells/10^6^ PBMCs or as a percent change between postvaccination and prevaccination frequencies. Intergroup differences were assessed using the Kruskall-Wallis test and within group differences using the Wilcoxon sign-ranked test, the nonparametric equivalent of the paired *t*-test. Post-vaccination changes within individual groups (compared to no change of 100%) were assessed using the sign rank test. Associations between study variables and percent change in frequencies of IFN-*γ*-expressing PBMCs (log_e_ transformed) were evaluated using multiple linear regression. Results of linear regression were back-transformed to provide fold-change in geometric means. All analyses were done using Stata, version 10 (Statacorp, College Station, TX, USA). 

## 3. Results

### 3.1. Study Population

A total of 48 children that fulfilled the study eligibility criteria were vaccinated. Baseline characteristics of the 48 individuals are shown in Table 1. Baseline age and nutritional status did not differ between the study groups. There were significantly more males in Group 1 compared to Groups 2 and 3 (*P* = 0.003). Pretreatment median infection intensities with *A. lumbricoides* were moderate in Groups 1 and 2. A high proportion of subjects in Groups 1 and 2 were infected with *T. trichiura* (80.0% versus 84.6%, resp.) but infection intensities were low. A surprising finding was that 53.3% of children in Group 3 were infected with *T. trichiura* despite having received repeated doses of albendazole over the previous 12 months. One individual in each of Groups 1 (5.0%) and 3 (6.7%) were infected with hookworm.

### 3.2. Adverse Reactions to Oral BCG

Adverse reactions were monitored weekly for 28 days after vaccination. Only mild adverse reactions were reported; headache (total of 15 episodes), fever (2), sore throat (1); diarrhea (3); changes in cervical lymphadenopathy (all 48 subjects had palpable cervical nodes before vaccination but no changes were noted over 28 days of observation).

### 3.3. Frequencies of IFN-*γ*-Expressing PBMCs

**Figure 2:**
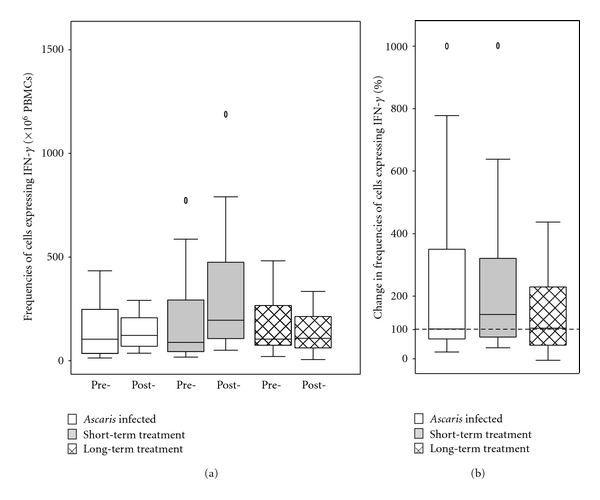
Frequencies of PPD-stimulated PBMCs expressing IFN-*γ* before and after vaccination with oral BCG Moreau. Frequencies were measured by ELISPOT. Graphs are (a) frequencies of PBMCs before (pre-) and after (post-)vaccination in active infection (clear), short-term anthelmintic treatment (grey), and long-term anthelmintic treatment (hatched) study groups; (b) percent changes in frequencies postvaccination compared to prevaccination. 100% shows no change. Shown are box plots with median (central line), interquartile range (box margins), 95% confidence intervals (bars), and outlying values (circles).

Data from 1 subject were excluded from the analysis because of a failure to produce detectable IFN-*γ*-expressing PBMCs to any of stimuli including mitogen after vaccination. PHA-induced frequencies of IFN-*γ*-expressing PBMCs were similar between the three groups before and after vaccination. Pre- and postvaccination frequencies of IFN-*γ*-expressing PBMCs following stimulation with PHA were Group 1 (prevaccination median 1805.6 × 10^6^ PBMCs, IQR 1016.7–2000.0 versus postvaccination median 1180.0, IQR 553.3–2000.0), Group 2 (prevaccination median 1808.9, IQR 1555.6–2000.0 versus post-vaccination median 1634.4, IQR 1249.2–2000.0). and Group 3 (prevaccination median 1671.1, IQR 1102.2–2000.0 versus postvaccination median 1277.8, IQR 1108.2–1893.3). The frequencies of PPD-induced IFN-*γ*-expressing PBMCs did not change significantly within any of the 3 study groups after vaccination and did not differ between the 3 groups either before or after vaccination (Figure 2). Pre- and post-vaccination frequencies of IFN-*γ*-expressing PBMCs were Group 1 (prevaccination median 95.6 × 10^6^ PBMCs, IQR 26.3–238.9 versus postvaccination median 122.2, IQR 70.0–206.7), Group 2 (prevaccination median 102.2, IQR 57.8–306.7 versus postvaccination median 195.6, IQR 106.7–475.6), and Group 3 (prevaccination median 84.4, IQR 55.6–246.7 versus postvaccination median 104.4, IQR 57.8–208.9). Percent changes in frequencies comparing post- with prevaccination frequencies were: all subjects (median 104.5%, IQR 60.1–333.3%), Group 1 (median 95.4%, IQR 63.1–350.0%), Group 2 (median 140.4, IQR 67.9–320.1), and Group 3 (median 92.9%, IQR 50.0–170.9%). There was, therefore, no evidence for postvaccination boosting of IFN-*γ* responses in this study population, although a trend of postvaccination boosting in Group 2 that received short-term anthelmintic treatment was seen. To see if individuals with low IFN-*γ* responses to PPD before vaccination increased their responses after vaccination, we stratified prevaccination responses into low and high representing values below and above the 25th centile of values for all subjects prevaccination (or 44 IFN-*γ*-expressing cells per 10^6^ PBMCs). The proportions of subjects with low PPD responses prevaccination were Group 1 (8/20 or 40%), Group 2 (1/12 or 8%), and Group 3 (3/15 or 20%). Six of these 12 (50%) “low-responder” individuals responded to vaccination (5/8 in Group 1; 0/3 in Group 2, and 1/1 in Group 3) by becoming “high responders” after vaccination.

### 3.4. Factors Associated with Percent Change in Frequencies of IFN-*γ*-Expressing PBMCs

We did an exploratory analysis to see if any baseline variables were associated with percent change in frequencies of IFN-*γ*-expressing PBMCs. Univariate analyses showed significant association with age (Fold-change 1.59, 95% CI 1.04–2.42, *P* = 0.03) but not sex (FC 0.69, 95% CI 0.86–1.11), hemoglobin (FC 1.56, 95% CI 0.93–2,62, *P* = 0.09), body mass index (FC 1.42, 95% CI 0.74–2.74, *P* = 0.29), and presence of prevaccination infections with *T. trichiura* (FC 0.85, 95% CI 0.40–1.81, *P* = 0.68) and *A. lumbricoides* (FC 1.15, 95% CI 0.56–2.32, *P* = 0.70). After multivariate analyses that controlled for these factors, the effect of age (FC 1.16, 95% CI 0.90–1.51, *P* = 0.24) lost statistical significance.

## 4. Discussion

Infectious diseases are a major cause of death and morbidity in populations living in developing countries and vaccination is the most effective public health strategy to reduce this disease burden. Many of the vaccines in use or under development for preventing infectious diseases are or will be delivered via the oral route [3, 4] and the poor efficacy and immunogenicity of such vaccines in poor populations represents a major barrier to the success of public health initiatives aimed at reducing infectious diseases. Geohelminth infections represent a potentially important cofactor that may contribute to vaccine hyporesponsiveness in underprivileged populations [13–15] and is easily modifiable given the wide availability of highly effective and cheap treatments. In the present study, we investigated the effect of concurrent infections with ascariasis on the immune response to a single booster dose with oral BCG. We measured vaccine immune responses using IFN-*γ* responses to tuberculin. Our data provide evidence that a booster dose of oral BCG did not significantly enhance IFN-*γ* responses in this population overall (104.5% change in IFN-*γ*-expressing cell frequencies) but this effect did not appear to be associated with ascariasis because neither short nor long-term repeated treatments with albendazole had any significant effect on IFN-*γ* responses. The failure of oral BCG to boost IFN-*γ* responses at a dose that is highly immunogenic in adult volunteers from the United Kingdom [16] provides further evidence for an immunological barrier to mucosal immunization in tropical populations.

Two previous studies have provided evidence that geohelminth infections may interfere with immune responses to vaccines: (1) Ecuadorian children infected with *A. lumbricoides* (infection intensity >10,000 eggs per gramme of faeces) were randomized to receive either 2 doses of albendazole or placebo separated by 30 days and then vaccinated with a single dose of 5 × 10^8^ cfus of the live-attenuated oral cholera vaccine, CVD 103-HgR, a dose shown previously to be suboptimal in populations of low socioeconomic status [8]. Vibriocidal antibodies and cellular responses to cholera B-subunit (CT-B) were measured. The results provided some evidence that pretreatment of ascariasis before vaccination improved both vibriocidal antibody levels [13] and Th1 cytokine responses to CT-B [14], but in the case of vibriocidal antibody levels there was a significant interaction with ABO blood group [13]. (2) Ethiopian adults infected with geohelminths, and presumed not to have been vaccinated previously with BCG, were randomized to receive either two doses of albendazole or placebo separated by 1 month, and those that were Mantoux-negative post-treatment were vaccinated with parenteral BCG. Production of IFN-*γ* protein was measured in supernatant fluids from PBMC cultures and there was evidence for a significant postvaccination increase in IFN-*γ* production in the albendazole-treated group while no change was observed in the placebo group [15]. Similarly, *Heligmosomoides polygyrus* a natural and chronic infection of the mouse small intestine, was associated with impaired IFN-*γ* production to OVA following vaccination with a novel oral OVA-expressing *Salmonella* vaccine [12].

There is evidence, therefore, that intestinal helminth infections may interfere with immune responses to oral vaccines. The finding of no effect of concurrent ascariasis on IFN-*γ* responses to PPD following vaccination with oral BCG indicates that infections with *A. lumbricoides* alone are unlikely to explain impaired immunity to oral vaccines. Other factors such as poor nutrition and immune deficiency associated with concurrent infections (e.g., HIV, tuberculosis, and malaria) may contribute to immune hyporesponsiveness to an oral vaccine. However, none of the children in the present study had evidence of significant nutritional abnormalities: none had hemoglobin levels below 11 g/dL or BMI values of 2 standard deviations below the mean for age. Further, neither of these nutritional parameters appeared to be associated with frequencies of IFN-*γ*-secreting PBMCs. Similarly, coinfections with powerful suppressive effects on the immune response such as HIV, malaria, and tuberculosis were of very low prevalence in our study population. Other chronic enteric parasitic infections that could contribute to hyporesponsiveness to oral vaccines include *G. intestinalis* and *E. histolytica*. Although not investigated in the present study, cysts of *G. intestinalis* and *E. histolytica/dispar *have been detected in 22.2% and 15.6%, respectively, of children aged 10 years living in the area where the study was conducted from a previous survey using standard microscopic detection in feces (Cooper et al., unpublished data). A significant proportion of children living in the rural tropics may have environmental enteropathy that has been associated with a histologic picture of blunted villi, abnormal crypt to villus ratio, and increased inflammatory cell infiltrate in the lamina propria [24, 25]. Environmental enteropathy is considered to be a major determinant of growth faltering in infants [26] living in unhygienic and unsanitary environments associated with intense exposure to enteric pathogens and may be contribute to a blunting of mucosal immunity [4]. Experimental animal infections with geohelminths are associated with the development of a Th2-mediated enteropathy [27], and there is evidence that geohelminth infections of humans may cause or contribute to histologic changes in the small intestine typical of environmental enteropathy [28].

We chose *A. lumbricoides* as the geohelminth infection of interest because previous surveys in the same area have shown that it is the most prevalent geohelminth present in our study population [21], because it resides in the small intestine where it may modify the mucosal immune response [19], which is also the primary site of attachment to and immune stimulation by oral vaccines, and because of our previous observations of the effects of concurrent ascariasis on the immune response to a live oral vaccine [13, 14]. We compared IFN-*γ* responses to PPD between 3 groups: children with active infections with *A. lumbricoides* (Group 1), and children who were infected but had received either short- (Group 2) or long-term (Group 3) treatments with albendazole prior to vaccination. The short-term treatment group was chosen to examine the short-term effects of *Ascaris* expulsion on vaccine responses: 2 doses of 400 mg of albendazole were given a month apart to ensure effective cure of ascariasis, to allow early recovery of the gastrointestinal mucosa, and to prevent the establishment of new infections due to migrating larvae. A trend of increased IFN-*γ*-secreting PBMCs was observed in Group 2 that received short-term anthelmintic treatment and a larger study might have shown a significant effect. The long-term treatment group was chosen to examine the long-term effects of maintaining the small intestine free of *A. lumbricoides* infections for a period of greater than a year. Such treatments should allow any long-term immunological or pathological effects of ascariasis on mucosal immune responses to be reversed if reversible. It is not clear why an effect on IFN-*γ* responses, if real, should have been observed for short but not long-term anthelmintic treatment.

Oral BCG Moreau RDJ was given routinely in Brazil until the 1970s when intradermal immunization was initiated to fall in line with the WHO EPI programme [17]. Overall, the safety data available from wide scale vaccinations in Brazil and Argentina indicate that oral BCG has an equivalent or superior safety profile compared to intradermal vaccinations [29, 30]. The immunological adjuvant effects of oral BCG and the ability to construct recombinant derivatives engineered to provide simultaneous vaccination to other mucosal pathogens [31] makes it an attractive vector for novel mucosal vaccines. Oral BCG Moreau RdJ has an excellent safety profile when administered at a dose of 100 mg and the risks are minimal [16, 17]. The only severe adverse event that has been associated with oral BCG Moreau RdJ in Brazil is suppurative adenitis, but with an extremely low frequency comparable to intradermal BCG. Minor adverse events associated with the vaccine include diarrhoea, vomiting, headache, sore throat, and cervical lymphadenopathy [16, 17]. Our observations from the present study support the excellent safety profile of this vaccine in a population of children living in the rural tropics.

## 5. Study Limitations

The study was designed as a pilot study to provide data that could be used to design an adequately powered randomized intervention study. The sample size was, therefore, small and we had limited power to detect significant effects. We measured the vaccine immune response to BCG using IFN-*γ* responses to PPD. Although PPD measures antimycobacterial immunity in general, an increase following oral BCG can be assumed to be due to enhanced immune responses to the vaccine itself and provides a reasonably robust method to measure postvaccination responses although such responses do not necessarily reflect enhanced protective immunity to *Mycobacterium tuberculosis*. Mucosal immune responses were measured at 28 days after vaccination at which time antigen-specific lymphocytes traffic between mucosal tissues in the systemic circulation [32]. The 28-day time point was based on the findings of a previous study that used the same vaccine as a booster dose in healthy volunteers in the UK, in a population that received BCG during childhood or adolescence and showed maximal IFN-*γ* responses for PPD at 1 and 3 months after vaccination [16]: the mean rise in IFN-*γ* frequencies at 1 months was ~3-fold with similar prevaccination frequencies as observed in the present study (~100 × 10^6^ IFN-*γ*-expressing PBMCs/mL). An explanation for a failure to observe postvaccination boosting of IFN-*γ* responses could relate to the kinetics of trafficking of PPD-responsive lymphocytes in: (a) children with a “damaged” enteropathic gut; (b) children that received BCG at birth; (c) circumstances in which exposures to environmental mycobacteria are likely to be different. A possible explanation for the failure of anthelmintic treatment to boost IFN-*γ* responses to PPD was the persistence of *T. trichiura* infections in both treatment groups, although postvaccination frequencies or changes in frequencies did not appear to differ by prevaccination *T. trichiura* infection status. Repeated doses of albendazole, although highly effective against *A. lumbricoides*, were of limited efficacy against infections with *T. trichiura* in this study. Similar observations have been made previously for single [33] and multiple doses of albendazole [21]. Although *T. trichiura* resides in the large intestine and, to affect vaccine responses would have to mediate inhibitory effects at the site of the interaction between the intestinal mucosa and BCG in the small intestine, there is evidence that *T. trichiura* may have regulatory effects at distal sites [34]. Future studies could examine the potential effects of *T. trichiura* infection, specifically, on immune responses to oral vaccines. Although the dose of oral BCG used for prophylaxis against tuberculosis has varied in Brazil [17], a single dose of 100 mg has been used for individuals without a history of contact with tuberculosis for many years [17]. For experimental studies in humans, single or multiple 100 mg doses of oral BCG have been administered either with 2% sodium bicarbonate (to neutralize gastric acidity) immediately before [30] or with vaccine [16] or without sodium bicarbonate buffer [35]. Although oral BCG has proved to be immunogenic using both approaches [16, 30, 35], a possible explanation for our negative findings are effects of gastric acidity on vaccine immunogenicity.

## 6. Conclusion

Our data show that children living in the rural tropics failed to respond to a single booster dose of oral BCG Moreau RDJ with an increased IFN-*γ* response to PPD. This apparent barrier to mucosal immunization appeared to be independent of ascariasis and was not significantly affected by treatment of these infections before vaccination. Our data provide further evidence for a barrier to oral vaccination in populations living in circumstances of poverty in developing countries that poses a major hurdle to the strategy of oral immunization in such populations unless steps are taken to improve mucosal health. One such measure would be to give anthelmintic treatment before vaccination, but as the present study shows, this intervention alone may provide limited benefits.

## Figures and Tables

**Table 1:** Baseline characteristics of study children. Group 1: active infection with *A. lumbricoides*; Group 2: short-term anthelmintic treatment; Group 3: long-term anthelmintic treatment. BMI-body mass index. *2 doses of 400 mg albendazole over 1 month. ^‡^7 doses of 400 mg albendazole over 12 months. ^§^: results of stool sample collected before short-term treatment.

Variable	Group 1 (*N* = 20)	Group 2 (*N* = 13)	Group 3 (*N* = 15)
Age			
Median (range)	10 (8–13)	10 (8–14)	10 (8–13)

Sex			
Male/female	14/6	3/10	3/12

BMI			
Median (range)	16.3 (13.2–20.9)	16.4 (13.7–19.2)	16.3 (14.3–26.4)

Hemoglobin (g/dL)			
Median (range)	12.3 (11.3–14.0)	12.5 (11.3–14.3)	12.5 (11.5–13.3)
Anthelmintic treatment			
Short-term treatment*	No	Yes	Yes
Long-term treatment^‡^	No	No	Yes

Geohelminth infections			
Baseline^§^			
*A. lumbricoides *	100%	100%	6.7%
Intensity, median (range) epg	7,728 (1,633–72,998)	13,135 (2,627–36,920)	0 (0–19,099)
*T. trichiura *	80.0%	84.6%	53.3%
Intensity, median (range) epg	568 (0–18,744)	213 (0–5,893)	0 (0–1,633)
Hookworm	5.0%	0%	6.7%

Postvaccination			
*A. lumbricoides *	85.0%	0%	0%
Intensity, median (range) epg	11,041 (0–135,965)	0 (0-0)	0 (0-0)
*T. trichiura *	85.0%	75.0%	21.4%
Intensity, median (range) epg	604 (0–3,976)	142 (0–9,940)	0 (0–710)
Hookworm	5.0%	0%	0%
